# Cross-sectional imaging in an inferior vena cava agenesis associated with antiphospholipid syndrome, lower limb, and portomesenteric deep vein thrombosis and renal insufficiency in a young patient: A rare presentation

**DOI:** 10.1016/j.radcr.2026.07.067

**Published:** 2026-08-08

**Authors:** Wany Linda Sami, Patient Aganze Migabo, Cristina Jbanca Florina

**Affiliations:** a Department of Radiology, Josephine Baker/Gonesse Hospital, Gonesse, France; b Hôpital Provincial Général de Référence de Bukavu (HPGRB), Faculty of Medicine, Université Catholique de Bukavu (UCB), South Kivu, Democratic Republic of the Congo

**Keywords:** Inferior vena cava agenesis, Anti-phospholipid syndrome, Porto-mesenteric and lower-limb thrombosis, Leg ulcers, KILT syndrome, Cross-sectional imaging

## Abstract

Inferior vena cava (IVC) agenesis is a rare congenital anomaly (prevalence 0.0005%-1%) often asymptomatic but can be complicated by deep vein thrombosis, particularly in the presence of acquired thrombophilia. There are 2 major presentations reported in literature, infrahepatic IVC agenesis associated mostly with lower-limb thrombosis and intrahepatic IVC agenesis associated frequently with splanchnic thrombosis and pulmonary embolism. We report the case of a 38-year-old man presenting with chronic bilateral leg ulcers evolving for 3 years. Cross-sectional imaging demonstrated partial agenesis of intra-hepatic IVC and complete agenesis of the infra-hepatic IVC up to the iliac bifurcation, a massive compensatory collateral network involving the azygos vein, hemiazygos, lumbar, and abdominal parietal systems, as well as multiple venous thrombosis of the portal vein, mesenteric vein, azygos vein and lower-limbs. The iliac veins drained via ascending lumbar veins to the hypertrophied azygos system. Morphology CT analysis showed a focal right renal infarct, but no atrophy was noted. Laboratory workup revealed a probable antiphospholipid syndrome (APS) and severe antithrombin III deficiency (22%), nephrotic syndrome, and alteration in glomerular filtration. This case illustrates the importance of recognizing radiological signs of IVC agenesis, particularly in the presence of atypical thrombosis or unexplained leg ulcers. The association with probable APS, multiple visceral thrombosis and renal function altered, makes this case exceptionally rare and educationally important.

## Introduction

Inferior vena cava (IVC) agenesis represents a rare congenital anomaly of the venous system with a prevalence ranging from 0.0005% to 1% in the general population [1], but reaching 4.2%-19.6% in young patients presenting with deep vein thrombosis (DVT) in some studies which considered patients with severe symptoms [[1], [2], [3]].

This malformation, often asymptomatic, can manifest by severe thromboembolic complications requiring early recognition to optimize management.

The association with antiphospholipid syndrome (APS) considerably amplifies thrombotic risk, favoring multiple, recurrent thrombosis affecting unusual vascular territories [[4], [5], [6]].

Renal abnormalities are frequently associated, formalized under the acronym “KILT syndrome” (kidney abnormalities, inferior vena cava agenesis, lower-limb deep vein thrombosis), including unilateral renal agenesis, hypoplasia, or ectopic kidneys [7].

Diagnosis relies on vascular imaging, with contrast-enhanced computed tomography (CT venography) as the noninvasive reference modality. MRI represents an excellent alternative avoiding radiation exposure or contraindications to contrast injection [[8], [9]]. Early recognition allows initiation of appropriate anticoagulation, generally prolonged, and screening for associated malformations [7,10].

This anomaly should be systematically sought in any young patient presenting with proximal DVT, particularly if bilateral or recurrent [6,11].

## Case presentation

Male patient, 38 years-old with no known medical history. He presented chronic bilateral leg ulcers evolving for 3 years without exploration or medical management. He came to the emergency department for exacerbation of pain in the ulcers.

On admission to the emergency department, he presented with a fever of 38°C, a very extensive ulcer on the anterior aspect of the right leg measuring approximately 20 cm, and another very extensive ulcer on the medial aspect of the left leg extending over approximately 15 cm.

Laboratory tests on admission revealed a biological inflammatory syndrome with CRP at 46 mg/L (0-3) without leukocytosis; anemia at 8.4 g/dL (NV: 13.0-18.0) normocytic, normochromic, aregenerative; thrombocytosis at 521 × 109/L (NV: 150-450); hypoalbuminemia at 17 g/L (Normal value (NV): 34-50); and stage III renal failure with creatinine at 142 µmol/L (65-104) and GFR by CKD-EPI (mL/min/1.7 m²) at 54 (NV: >90).

During hospitalization, additional laboratory tests were performed and revealed nephrotic syndrome, severe antithrombin III deficiency (22%) (NV: 80%-120%). Elevated levels of antiphospholipid antibodies (lupus anticoagulant and β2-glycoprotein Immuno-globulin G), prolonged activated partial thromboplastin time with circulating anticoagulant, strongly positive anti-B2GP1 IgG, positive DNA, and positive ANA (AntiNuclear Antibodies) at 1/80 speckled pattern suggesting probable primary antiphospholipid syndrome.

The patient was lost to follow-up; consequently, the confirmatory serological testing at 12 weeks, required to formally establish the diagnosis of antiphospholipid syndrome according to ACR/EULAR (American College of Rheumatology/European Alliance of Associations for Rheumatology) classification criteria, could not be performed.

Chronic low-activity hepatitis B was discovered without hepatic function impairment. Abdominopelvic CT scan was performed on admission for undocumented renal failure, which did not reveal urinary obstructive syndrome. Nevertheless, it revealed multiple varicose, superficial venous collateral pathways in the abdominopelvic wall and multiple bilateral iliac and inguinal lymph nodes.

For better analysis, abdominopelvic and lower-limb CT angiography were performed on the sixth day of hospitalization; in spontaneous contrast, arterial phase, portal phase, and delayed phase after injection of iodinated contrast medium (100 mL at 3.5 mL/s). It revealed partial agenesis of intrahepatic vena cava and complete agenesis of the infrahepatic vena cava from the confluence of the iliac veins to the hepatic veins. The length of the absent segment extends over approximately 216.4 mm (T12-L5). The compensatory collateral drainage network is ensured primarily by the azygos system, with the azygos vein being dilated and reaching a diameter of 22 mm (NV: less than 10 mm). It has a right retroperitoneal course along the spinal column. Lumbar and abdominal parietal collaterals veins were also noted. The common, external, and internal iliac veins were present and patent with slightly increased caliber with drainage via ascending lumbar veins.

Partial thrombosis of the left branch of the portal vein was visualized over a length of approximately 23 mm without evident portal cavernoma detected. The caliber of the portal vein was 16 mm higher than the normal value (13 mm). There was also associated partial thrombosis of the superior mesenteric vein extending to approximately 26 mm. Organ analysis noted essentially a parenchymal-sinusal defect of the right kidney with a “cortical rim ring” suggesting a focal infarction. Also, hepatic perfusion disturbance. Lower-limb exploration noted suspected deep vein thrombosis of the iliofemorotibial- fibular region on the right and left popliteal veins, significant liquid infiltration of subcutaneous soft tissues. Doppler ultrasound of the lower limbs was performed and revealed right femoropopliteal deep vein thrombosis from the origin of superficial femoral vein to the distal popliteal vein.

Renal involvement manifested as biological nephrotic syndrome characterized by massive proteinuria at 8 g/24 hours and severe hypoalbuminemia at 17 g/L (normal values 34-50 g/L). Renal biopsy revealed the presence of a fibrin thrombus on 1 glomerulus and at the level of an arteriole, without other glomerular abnormality, with immunofluorescence indicating minimal change glomerular lesions. There was no evidence of lupus on renal biopsy.

The evolution of renal function was favorable under treatment, with improvement in serum creatinine from 142 µmol/L on admission to 100-101 µmol/L, and in glomerular filtration rate (GFR) estimated by CKD-EPI from 54 mL/min/1.73 m² to 81-82 mL/min/1.73 m², allowing progression from stage III renal insufficiency to quasi-normal function.

On the 35th day of hospitalization, the patient presented intense abdominal pain, clinically suggesting mesenteric ischemia. Abdominopelvic CT angiography was performed and revealed partial thrombosis of the distal portion of the azygos vein at the mid-thoracic segment level (T6-T10) extending over approximately 85 mm in length but no mesenteric artery occlusion was noted or abnormally in bowel layer perfusion. However, acute colitis was described.

The anticoagulant therapeutic strategy was based on initial administration of low molecular weight heparin (LMWH) at curative dose, followed by vitamin K antagonist treatment (Coumadin 8 mg in the evening) with a target INR between 2 and 3, a therapeutic target that was achieved with this dosage. The choice of VKA was necessary in the particular clinical context associating probable antiphospholipid syndrome (APS) with positivity of lupus anticoagulant and strongly positive anti-β2- glycoprotein I IgG antibodies, as well as severe antithrombin III deficiency.

Management of chronic bilateral cutaneous ulcers required daily local care during hospitalization. The indication for skin grafting was established; however, the high anesthetic risk contraindicated surgical intervention, leading to favoring a directed healing strategy with prescription of daily outpatient dressings until complete healing.

**Fig. 1:**
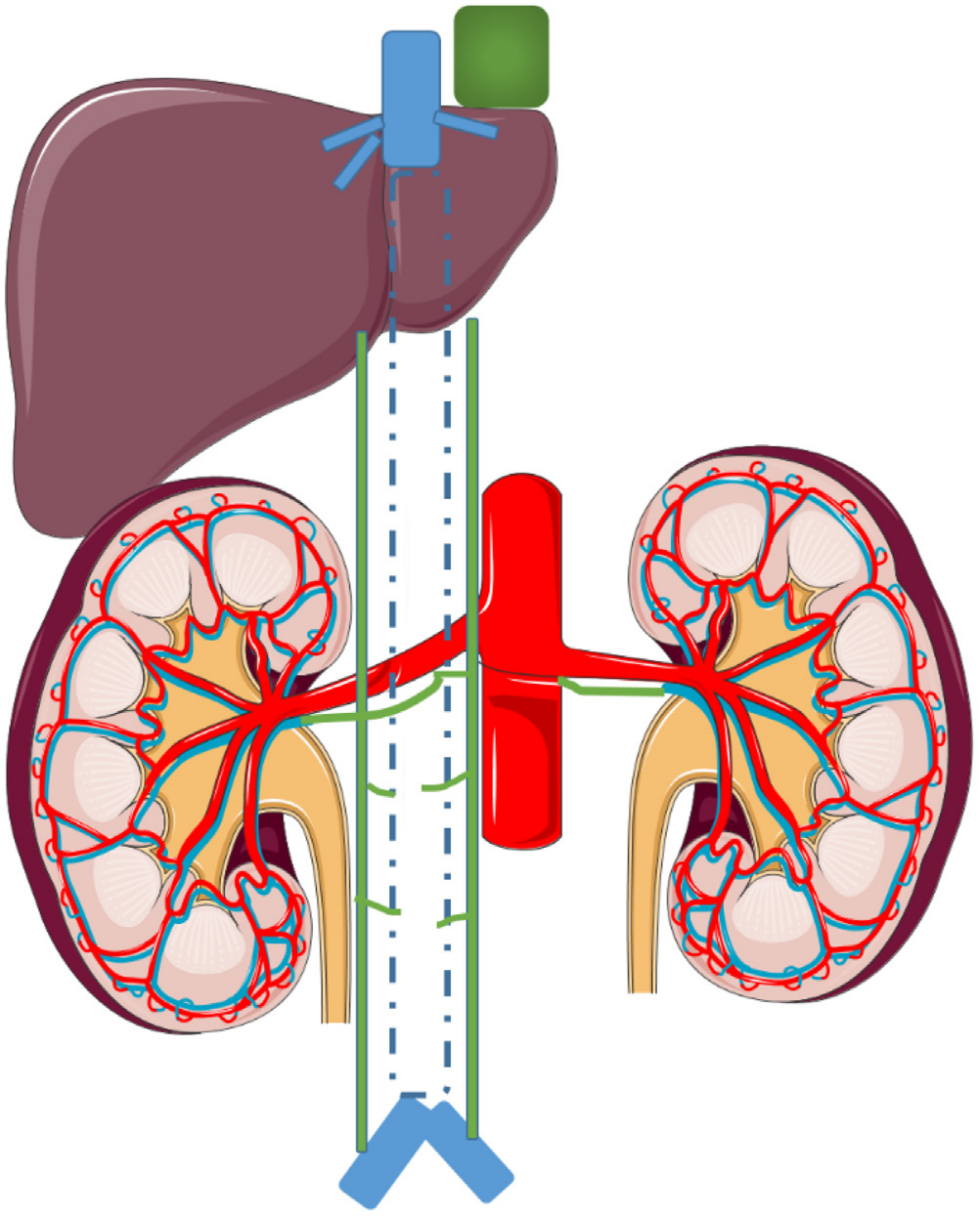
Schematic representation of IVCA and collateral pathways: In blue: normal venous network (iliac veins and a partial intrahepatic vena cava) Dotted blue: agenetic segment. In green: hypertrophied bypass pathways through the ascending lumbar veins to reach the « mega-azygos » vein which reroutes blood upward to the heart. Some anatomical elements were adapted from Servier Medical Art (https://smart.servier.com), licensed under CC BY 4.0 (https://creativecommons.org/licenses/by/4.0/).

**Fig. 2:**
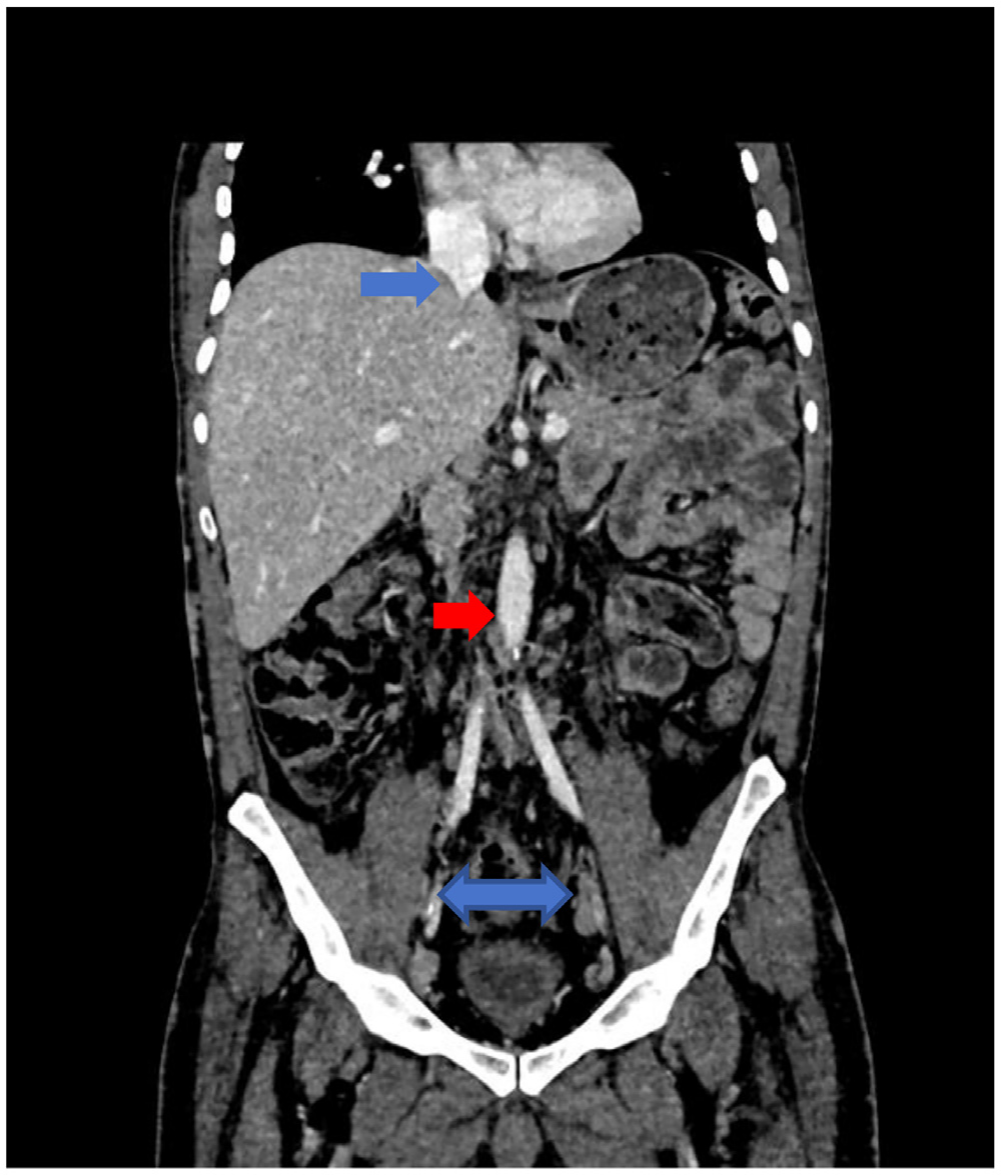
Abdomino-pelvic CT scan: Coronal plan through the IVC showing partial agenesis of intrahepatic segment and complete agenesis of infra-hepatic segment (blue arrow), normal abdominal aorta (red arrow) and iliac veins (blue double-headed arrow).

**Fig. 3:**
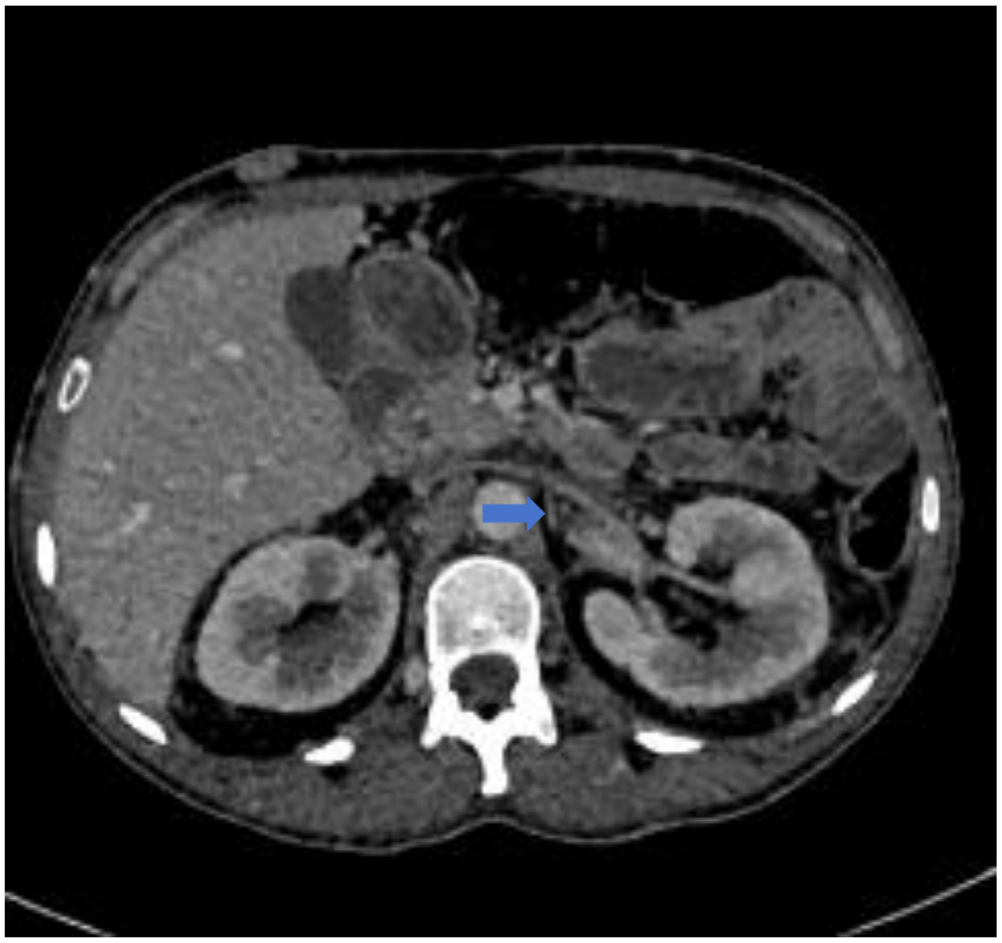
Abdomino-pelvic CT scan: Axial plan through the renal veins showing transaortic renal drainage (blue arrow).

**Fig. 4:**
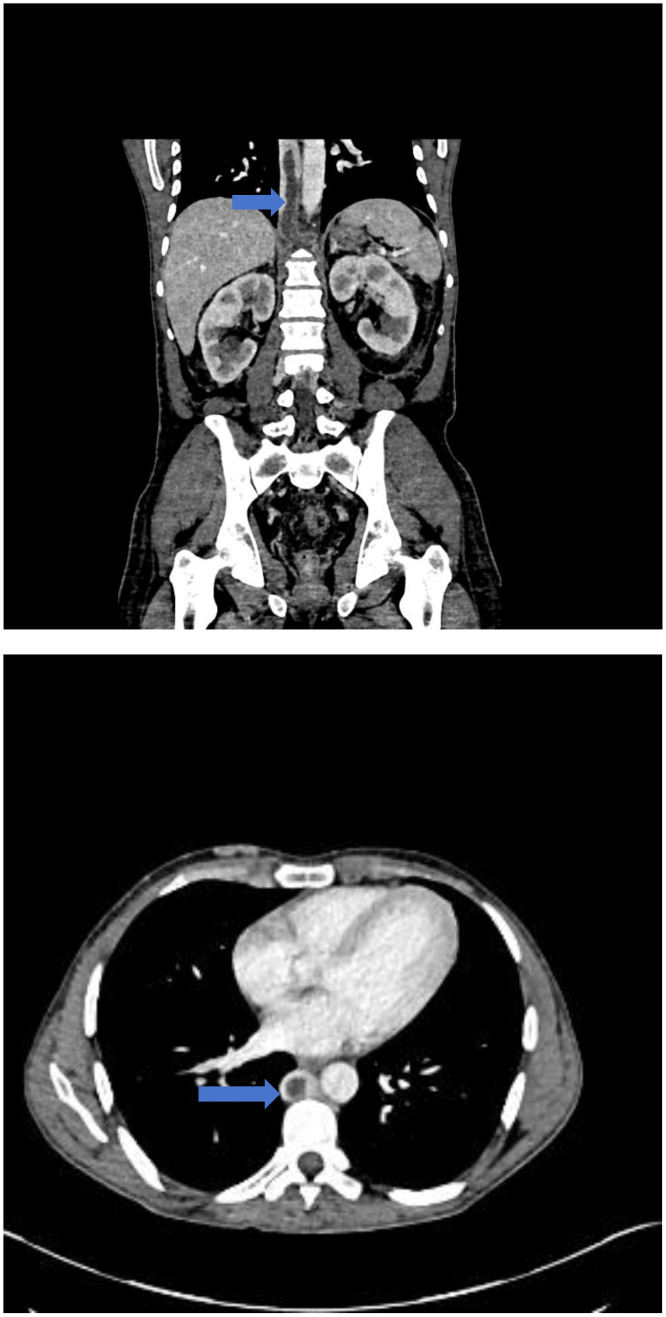
Abdomino-pelvic CT scan: Coronal and axial plans showing an enlarged azygos vein with thrombus (Blues arrows).

**Fig. 5:**
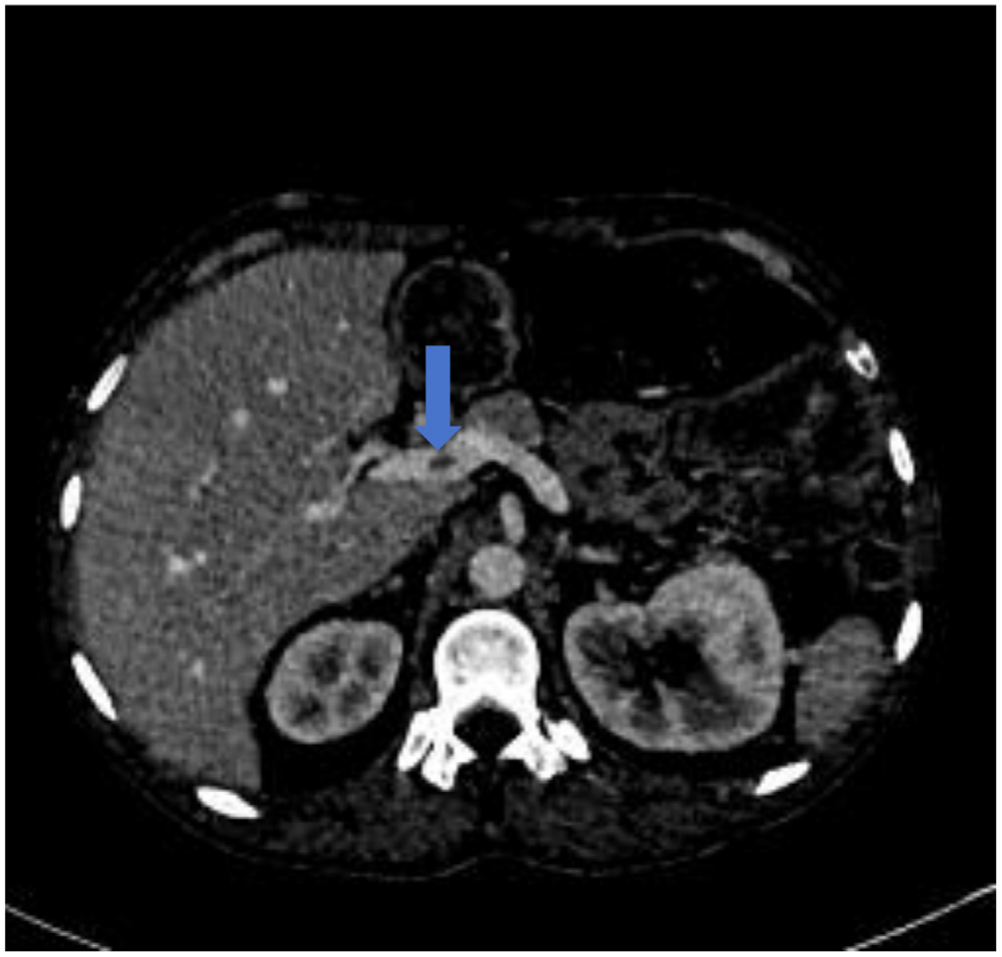
Abdomino-pelvic CT scan: Axial plan showing a thrombus within the porta vein (Blue arrow).

From a nephrological standpoint, the nephrotic syndrome with minimal change glomerular lesions, confirmed by immunofluorescence on renal biopsy, justified the initiation of corticosteroid therapy at 1 mg/kg, 60 mg per day of prednisone, with planed nephrological follow-up (Fig. 1, Fig. 2, Fig. 3, Fig. 4, Fig. 5).

## Discussion

Inferior vena cava (IVC) agenesis constitutes a rare congenital vascular anomaly with a prevalence in the general population reaching 1% [1], but can reach up to 2% in young patients (<30 years) presenting with deep vein thrombosis (DVT), and even more when we consider a group of young patients with severe symptoms 4.2%-19.6% [1,2].

Our case illustrates an exceptionally rare presentation associating partial intrahepatic and infrahepatic IVC agenesis, probable antiphospholipid syndrome (APS), multiple visceral venous thrombosis (portal, mesenteric, and azygos) as well as lower-limb thrombosis, and chronic bilateral leg ulcers evolving for 3 years.

The embryological development of the IVC represents a complex process; aberrations in this developmental process lead to different forms of congenital anomaly, among others agenesis of infrahepatic segments [12,13]. The latter suggests a developmental defect proper of the 3 pairs of primitive veins (posterior cardinal, subcardinal, and supracardinal) [9], which also applies to our context additionally to partial agenesis of intrahepatic portion developed from right vitellin vein.

Analysis of imaging data by CT angiography formally confirms the diagnosis of congenital agenesis of the inferior vena cava (IVC) and allows exclusion of chronic venous thrombosis based on several major differential criteria. The complete absence of parietal enhancement or pericaval soft tissue at the level of the absent segment constitutes a sign of congenital agenesis [3,8,11,14], whereas chronic thrombosis is typically accompanied by parietal enhancement reflecting thrombus organization or an inflammatory reaction [8,14]. The total absence of intraluminal thrombotic material or calcifications within the absent segment on computed tomography sections is unfavorable to chronic thrombosis [3,8,11,14,15]. The hypothesis of post-thrombotic atresia is ruled out because of the absence of a residual fibrous cord or pericaval scar on imaging [3,8,11,15].

The presence of a massive, bilateral, and perfectly symmetrical compensatory collateral network, mainly involving the hypertrophied azygos system (diameter of 22 mm, normal value <10 mm) and the ascending lumbar veins, represents a cardinal element of agenesis [[3], [9], [13]], contrasting with the more variable and less organized collaterals observed in thrombosis [8,14]. The patency of the common, external, and internal iliac veins with drainage via the ascending lumbar veins to the azygos system [[8], [9], [12]], associated with the presence of an anatomical variant of the renal veins [[9], [13]], supports the congenital origin of the anomaly.

Cases have been described in the literature with acute bilateral deep vein thrombosis in young subjects revealing inferior vena cava agenesis in young patients [14,15]; our patient presented an unusual clinical evolution dominated by chronic bilateral leg ulcers evolving for 3 years without prior exploration in a region where imaging modalities are easily access.

This atypical presentation probably reflects severe chronic venous insufficiency secondary to prolonged venous stasis in the collateral system [15], which also led to right femoro-popliteal venous thrombosis in our case.

Chronic venous ulcers as a mode of revelation of IVC agenesis remain poorly described in the literature, making this case educationally important.

Also, the association of IVC agenesis with probable APS represents an exceptionally rare clinical situation creating major thrombotic risk through the combination of 2 mechanisms: chronic venous stasis related to anatomical anomaly and acquired hypercoagulability secondary to probable APS [4,5,16]. A literature review identifies fewer than 10 similar documented cases [3].

Abnormal renal function was scarcely reported, at least renal hypotrophy was noted in KILT syndrome (kidney and IVC abnormalities with leg thrombosis) without impairment of renal function [17], this raised question about acquired KILT syndrome secondary to renal vein drainage insufficient. However, most cases of KILT syndrome didn't mention biochemical function renal tests abnormalities as for our patient who did not present a major renal morphological anomaly, functional renal involvement was significant with stage III renal failure and nephrotic syndrome (proteinuria 8 g/24 hours, albuminemia 17 g/L). This presentation partially evokes “KILT syndrome” [7].

The frequent association between IVC agenesis and renal abnormalities (up to 60% of cases) is explained by the common embryological origin of the venous and urinary systems during organogenesis [13]. In our case, renal infarction and nephrotic syndrome seem more related to the severe thrombophilic context and probable APS than to a primitive congenital renal malformation [5,16].

In our observation, initial laboratory workup revealed APS by positivity of lupus anticoagulant and strongly positive anti-β2-glycoprotein I IgG antibodies, associated with severe antithrombin III deficiency (22%, normal 80%-120%).

The severe antithrombin III deficiency in our case is probably the result of the combination of mainly 2 mechanisms: excessive consumption by massive thromboses and deficiency related to nephrotic syndrome. The hereditary origin of this deficiency is less probable; the patient does not report any history of specific pathology or even previous biological workups, prior to his episode of chronic lower-limb ulcers over 3 years, which could confirm this hypothesis.

The hypothesis related to the influence of anticoagulant treatment is also improbable, because the patient affirms the absence of any management before his admission, and anticoagulant treatment during his hospital stay was initiated after the biological workup.

All of these mechanisms explain the occurrence of multiple and extensive venous thrombosis affecting unusual vascular territories.

The simultaneous presence of portal, mesenteric, and azygos vein thrombosis constitutes a remarkable element of our observation. Portal and mesenteric thrombosis associated with IVC agenesis are rarely reported in the literature [3,10,18]. Pons et al. described a similar case associating portal thrombosis and mesenterico-portal confluence, APS, and concomitant IVC agenesis [18].

Azygos vein thrombosis, occurring secondarily on 35th day of hospitalization, has particular significance in this context since the azygos constitutes the main collateral pathway for venous drainage [4,9].

Radiological recognition of IVC agenesis is of capital importance to avoid diagnostic errors and guide management [10]. The main pitfalls include confusion with extensive chronic IVC thrombosis, lack of awareness of indirect signs (azygos hypertrophy, paravertebral collaterals), and omission of complete thrombophilic workup. In our case, the decisive radiological criteria were complete agenesis of the caval segment with well- developed symmetrical collaterals, azygos hypertrophy (22 mm), absence of parietal enhancement, presence of an anatomical variant of the renal veins.

Our observation presents several elements of major educational interest including the exceptionally rare association of IVC agenesis, probable APS, and multiple visceral thrombosis; the atypical clinical presentation with chronic leg ulcers delaying diagnosis by 3 years; complete radiological demonstration of collateral anatomy and multiple thrombosis; secondary occurrence of azygos vein thrombosis (main collateral) complicating clinical evolution; and renal involvement combining infarction and nephrotic syndrome related to severe thrombophilic context and probable APS rather than primitive congenital renal malformation.

To our knowledge, this specific combination has been rarely reported in the literature [3,11].

## Conclusion

This case illustrates an exceptionally rare and complex presentation of partial intra-hepatic and infra-hepatic inferior vena cava agenesis associated with probable antiphospholipid syndrome and multiple visceral venous thrombosis (portal, mesenteric, and azygos). Key radiological findings include complete agenesis of the IVC in its normal anatomical position, a massive compensatory collateral system via hypertrophied azygos-hemiazygos, and extensive thrombosis affecting multiple vascular territories.

Key messages for radiologists are to systematically search for IVC agenesis in any azygos hypertrophy (>10 mm), developed lumbar and parietal collateral veins and to perform complete thrombotic workup including porto-mesenteric territories in case of confirmed IVC agenesis, and to alert the clinician to the major thrombotic risk requiring prolonged anticoagulation.

This case underscores the central role of the radiologist in early recognition of this rare but clinically significant entity, allowing initiation of appropriate management and prevention of potentially serious complications.

## Author contributions

SLW drafted the manuscript, including the case description and its conceptualization. JFC, co-author, was responsible for study conceptualization, writing and final validation. MAP, the corresponding author supervised the manuscript drafting including images set up, contributed to the editing and performed the final revisions. All authors approved the final version of the manuscript for publication.

## Ethical approval

This case report was conducted in accordance with the principles of the Declaration of Helsinki. As per institutional guidelines for case reports, formal ethical approval was not required.

## Data availability

The data supporting the findings of this case report are available from the corresponding author upon reasonable request.

## Patient consent

Informed consent for publication of this case report and associated images was obtained from the patient.

## References

[1] Simon R.W., Amann-Vesti B.R., Pfammatter T., Koppensteiner R. (2006). Congenital absence of the inferior vena cava: a rare risk factor for idiopathic deep-vein thrombosis. J Vasc Surg.

[2] Schneider J.G., Eynatten M.V., Dugi K.A., Duex M., Nawroth P.P. (2022). Recurrent deep venous thrombosis caused by congenital interruption of the inferior vena cava and heterozygous factor V Leiden mutation. J Intern Med.

[3] Saab K., Brahmandam A.S., Brackett A.L., Desai M.M., Dardik A., Guzman R.J. (2023). Systematic review of inferior vena cava atresia. J Vasc Surg Ven Lymph Disord.

[4] Spooner J.T.R., Ginting N., Birk M. (2020). Congenital absence of inferior vena cava in a 33-year-old with antiphospholipid antibody syndrome: a case report. UBC Med J.

[5] Higa M., Kojima M., Ohnuma S., Hamanaka S., Yamamuro W., Sugiura H., et al. Portal and mesenteric vein and inferior vena cava thrombosis associated with antiphospholipid syndrome. 2001 https://europepmc.org/article/MED/11813853 (Accessed July 1, 2026). 10.2169/internalmedicine.40.1245 11813853

[6] Erok B, Win N, Agolli E, Olcay A. A common manifestation with an uncommon diagnosis: intractable deep vein thrombosis due to IVC agenesis in a young woman, 2021 https://journals.sagepub.com/doi/full/10.1177/26324636211038081 (Accessed July 1, 2026).

[7] Moragón-Ledesma S., Galeano-Valle F., Calleja-Cartón E., Del-Toro-Cervera J., Demelo-Rodríguez P. (2020). Bilateral Deep Vein Thrombosis, Vena Cava Agenesis, and Renal Abnormalities: KILT Syndrome-A Case Report and Literature Review. J Cardiovasc Transl Res.

[8] Koc Z., Oguzkurt L. (2007). Interruption or congenital stenosis of the inferior vena cava: prevalence, imaging, and clinical findings. Eur J Radiol.

[9] Edward Bass J, Redwine MD, Kramer LA, Huynh PT, Harris JH. Spectrum of congenital anomalies of the inferior vena cava: cross-sectional imaging findings. Radiographics 2000, 20 (3). https://pubs.rsna.org/doi/abs/10.1148/radiographics.20.3.g00ma09639?journalCode=radiographics (accessed July 1, 2026). 10.1148/radiographics.20.3.g00ma09639 10835118

[10] Cruz I.E., Ferreira P., Silva R., Silva F., Madruga I. (2019). Inferior Vena cava agenesis and deep vein thrombosis: a pharmacological alternative to vitamin K antagonists. Eur J Case Rep Intern Med.

[11] M Lambert, P Marboeuf, M Midulla, N Trillot, J-P Beregi, C Mounier-Vehier, et al. Inferior vena cava agenesis and deep vein thrombosis: 10 patients and review of the literature, 2010. https://journals.sagepub.com/doi/10.1177/1358863X10391355?__cf_chl_f_tk=hX4J6jFzOTLApHSNgCNI8xUsxs0sB78Vq9327N9fNqY-1782886569-1.0.1.1-rC57M6gyKhlYx8Om7KDbXKr5D7fFDv3f9dIHw2OWAs4 (Accessed July 1, 2026). 10.1177/1358863X10391355 21183652

[12] Bass J.E., Redwine M.D., Kramer L.A., Harris J.H. (1999). Absence of the infrarenal inferior vena cava with preservation of the suprarenal segment as revealed by CT and MR venography. Am J Roentgenol.

[13] Spentzouris G., Zandian A., Cesmebasi A., Kinsella C.R., Muhleman M., Mirzayan N. (2014). The clinical anatomy of the inferior vena cava: a review of common congenital anomalies and considerations for clinicians. Clin Anat.

[14] Morosetti D., Picchi E., Calcagni A., Lamacchia F., Cavallo A.U., Bozzi A. (2018). Anomalous development of the inferior vena cava: case reports of agenesis and hypoplasia. Radiol Case Rep.

[15] Parsa P, Lane III JS, Barleben AR, Owens EL, Bandyk D. Congenital agenesis of inferior vena cava: a rare cause of unprovoked deep venous thrombosis. Semant Schol n.d. https://www.semanticscholar.org/paper/Congenital-agenesis-of-inferior-vena-cava%3A-a-rare-Parsa-Lane/6e74ec22ed6175490cff01a36a114edbfaf4782d (Accessed July 1, 2026). 10.1016/j.avsg.2015.01.003 25747887

[16] Berrady R, Khammar Z, Lamchachti L, Lahlou M, Rabhi S, Bono W. Thrombosis of the inferior vena cava revealing primary antiphospholipid syndrome: a case report] J Mal Vasc. 2010 ;35(1):47-50. doi:10.1016/j.jmv.2009.11.004. 2009. (Accessed July 1, 2026). 10.1016/j.jmv.2009.11.004 19969435

[17] Fung J.K., Yeung V.H.W., Chu S.K., Man C.W. (2017). KILT (kidney and IVC abnormalities with leg thrombosis) syndrome in a 41-years-old man with loin pain and fever. Urol Case Rep.

[18] Pons L.L., Vizcarro N.C., Borrell S.L., Abbad C.M. (2017). Mesenteric-portal axis thrombosis and deep venous thrombosis in a patient with inferior vena cava agenesis. Revista Espanola de Enfermedades Digestivas.

